# Autochthonous and Travel Acquired Hepatitis E Virus in Australia

**DOI:** 10.3389/fmicb.2021.640325

**Published:** 2021-02-04

**Authors:** Jacinta O’Keefe, Lilly Tracy, Lilly Yuen, Sara Bonanzinga, Xin Li, Brian Chong, Suellen Nicholson, Kathy Jackson

**Affiliations:** Victorian Infectious Diseases Reference Laboratory, Royal Melbourne Hospital, Doherty Institute for Infection and Immunity, Melbourne, VIC, Australia

**Keywords:** hepatitis E virus, Australia, phylogenetics analysis, autochthonous, zoonotic

## Abstract

**Background:**

Hepatitis E virus (HEV) is a common cause of acute viral hepatitis with significant morbidity and mortality, particularly in pregnant women. There are four major genotypes which can cause disease in humans. Genotypes 1 and 2 are usually associated with outbreaks and spread via facal/oral route or contaminated water. Genotypes 3 and 4 are zoonotic and usually associated with handling of pigs or consumption of contaminated pork. The strains circulating in Australia have never been characterized.

**Rationale/Aims:**

The aims for this project are to identify the HEV genotypes found in Australia and link them to possible sources of transmission by phylogenetic analysis.

**Materials and Methods:**

Between 2015 and 2020, 91 HEV isolates were sequenced and genotyped using an in-house PCR. Sixty-six of these were also sequenced by using the international HEVnet primers. Genotypes were determined using the BLASTn program. Relatedness to other strains in Australia was determined by phylogenetic analyses of the HEVnet sequences. Isolates were also stratified by state of origin, gender, age, predisposing factors and travel history (if known).

**Results:**

Of the 91 HEV isolates sequenced, 55 (60.4%) were genotype 1. There were 34 (37.4%) genotype 3 strains and two genotype 4 (2.2%). At least 20 of the genotype 1 strains have been linked to travel in India, and another three with Pakistan. Five of the “Indian” strains were closely related and are suspected to have originated in Gujarat. Phylogenetic analysis also showed that 12 genotype 3 strains were genetically related and potentially acquired in/from New South Wales, Australia. The two genotype 4 strains may have originated in China.

**Discussion:**

This is the first study to describe the HEV isolates identified in Australia. The results infer that HEV may be acquired during overseas travel as well as locally, presumably from consumption of pork or pork-related products. The phylogenetic analyses also reveal clusters of infection originating from India and Pakistan. This study provides some insight into the source and epidemiology of HEV infection in Australia which may be used to guide public health procedure and enable the implementation of measures to deal with potential outbreaks of infection.

## Introduction

Hepatitis E virus (HEV) is a major cause of acute hepatitis worldwide and is associated with significant morbidity and mortality in pregnant women and people with chronic liver disease or immunosuppression. Previously, HEV was thought to predominantly circulate in developing countries, causing an estimated 3.4 million symptomatic cases and 70,000 deaths annually (Rein et al., 2012), with cases in industrialized countries linked to travel in HEV endemic regions. In the last decade, mounting evidence has demonstrated that zoonotic HEV is endemic in many developed countries including France, England, Bulgaria and Japan, and it is estimated that there are as many as two million cases of autochthonous HEV in Europe annually (European Association for the Study of the Liver, 2018).

HEV is a small, non-enveloped, RNA virus with a positive-sense 7.2kb genome containing a 5′ untranslated region (UTR), three open-reading frames (ORF) and a polyadenylated 3′ UTR. ORF1 encodes a non-structural protein, ORF2 encodes a capsid protein of 660 amino acids and ORF3 encodes a small phosphoprotein, which enables release of virions from hepatocytes (Nagashima et al., 2011). As a member of the *Hepeviridae* family (Lu et al., 2006), HEV is classified as a species of *Orthohepevirus A* in the *Orthohepevirus* genus. Recent revisions have proposed eight genotypes and 36 subtypes that infect mammals, with genotypes 1-4 being a major cause of disease in humans (Smith et al., 2020).

The epidemiology, clinical presentation and reservoir of HEV can vary depending on the viral genotype. HEV-1 and -2 exclusively infect humans and are transmitted by the faecal-oral route via contaminated water in low-middle-income countries within Africa and Asia, typically causing symptomatic infection in patients aged 15–40 years (Kamar et al., 2014). Large outbreaks of HEV-1 infection are most common in South and East Asia and, whilst HEV-2 is less prevalent, it has been linked to outbreaks in Mexico, Nigeria and Namibia (Maila et al., 2004; Lu et al., 2006).

In contrast, HEV-3 and -4 are highly diverse, zoonotic viruses that have been isolated worldwide in humans and animals including wild boar, pigs and deer. Transmission in humans is primarily associated with the consumption of contaminated, undercooked pork or game (wild-caught) meat but has also been linked to shellfish consumption. In addition, transfusion-transmission has been reported for HEV-3 infection (Hewitt et al., 2014; Forgan-Smith and Macdonald, 2019). Whilst HEV-3 is thought to be asymptomatic in up to 98% of cases, it is associated with symptomatic disease in older men or people with liver damage (Kamar et al., 2014) and can cause chronic infection in immunosuppressed patients, which can lead to cirrhosis (Kamar et al., 2008a,b). HEV-3 has been reported in multiple countries on six continents (excluding Antarctica). HEV-4 has been found in human and swine populations in mostly Asian countries (Lu et al., 2006; Zhu et al., 2014) but has also been detected in France (Colson et al., 2012) and Switzerland (Fraga et al., 2018).

There is limited data about the genotype distribution of HEV in Australia or the incidence of autochthonous HEV. Hepatitis E has been a notifiable disease in Australia since 1999 and, over the last 10 years, approximately 30–60 cases of Hepatitis E infection have been reported to the Commonwealth Department of Health annually, with 250 total cases reported between 2015 and 2020 (Australian Government Department of Health, 2020). The HEV seroprevalence in Australian blood donors has been reported as 5.9% (Shrestha et al., 2014), which is comparable to New Zealand (9%) (Hewitt et al., 2018) and Scotland (6.1%) (Thom et al., 2018) and lower than that seen in South Western France (52%) and The Netherlands (30%). In Australia, most cases of Hepatitis E infection have previously been ascribed to travel to a HEV endemic region (Gunaratnam et al., 2014). However, locally acquired infection with HEV-3 does occur, with a previous report of HEV transmission by blood transfusion (Speers et al., 2015) and a small outbreak in 2013/4 linked to consumption of undercooked pork at a New South Wales (NSW) restaurant (Yapa et al., 2016). Phylogenetic analysis was used to genotype a single HEV-3 isolate in a 2016 study investigating the prevalence of HEV RNA in Australian blood donations (Shrestha et al., 2016). However, there has not been any comprehensive phylogenetic analysis of Australian HEV isolates. This study aims to characterize the isolates of HEV positive samples sent to the Victorian Infectious Disease Reference Laboratory (VIDRL) from laboratories across Australia within the last 5 years and link them to possible sources of transmission by comparing the genetic relatedness of the isolates.

## Materials and Methods

### Patient Samples

VIDRL is a specialized laboratory that performs HEV molecular testing for most states and territories of Australia with the exception of Queensland (QLD). Serum samples may be sent specifically for primary or confirmatory HEV RNA testing, or referred from the VIDRL serology laboratory after a reactive IgG result is obtained. Ninety one HEV RNA positive samples tested at VIDRL between 2015 and 2020 were sequenced for genotype determination using an in-house PCR and Sanger sequencing as below. We were able to perform subsequent sequence analysis using the HEVNet primers on 66 of these and phylogenetic analysis. These 66 isolates (phyloanalysis population) were compared to demographic details and relevant clinical history of the patient. Ethics approval for this study was granted by the Melbourne Health Human Research Ethics Committee (HREC/62312/MH-2020).

### Serological Testing

Anti-HEV IgG was tested using the HEV ELISA 4.0 kit, (MP Biomedicals Asia Pacific Pte Ltd, Singapore, formerly Genelabs Diagnostics Pte) as per manufacturer’s instructions. The test results are expressed as the absorbance of the patient sample to the assay cut-off (s/co value). A sample with a s/co value greater than 1.0 is considered reactive and less than 1.0 is considered negative. As per VIDRL protocol, prior to 2018 serum/plasma samples with a s/co value of greater than 3 were automatically referred on for PCR testing, as these samples were considered more likely to reflect current HEV infection (Zaaijer et al., 1993). This practice was amended in 2018 to include all samples with reactive IgG.

### Molecular Testing

Serum or plasma samples for HEV PCR were stored at −70°C before assaying. RNA was extracted from 140 μL of serum with the QIAamp Viral RNA Mini Kit (QIAGEN, Hilden, Germany) as per manufacturer’s instructions. Between 2015 and 2020, 909 samples were screened for HEV RNA. The qualitative RealStar HEV RT-PCR Kit (Altona Diagnostics, Hamburg, Germany) was used from 2015 to 2018 and the quantitative RealStar HEV RT-PCR Kit 2.0 from 2018 onward.

For 91 HEV RNA positive samples, genotypes were determined prospectively by sequencing a 191bp fragment of the ORF2 in an in-house PCR as described previously (Erker et al., 1999) and using the BLASTn program on the NCBI BLAST web server.

Sixty-six of the 91 HEV positive serum samples were assayed in a two-step, nested RT-PCR using the HEVnet primers shared by RIVM (Rijksinstituut voor Volksgezondheid en Milieu, Nederlands) (Mulder et al., 2019). A 493 nt amplicon to the HEVORF2 region was generated with Superscript III Reverse Transcriptase (Invitrogen, Waltham, MA, United States) and HotStarTaq DNA Polymerase (Qiagen, Hilden, Germany) as previously described (Bruni et al., 2018). Purified PCR product was sequenced using ABI-Prism 3730 Genetic Analyser (Applied Biosystems, Life Technologies, Ltd, Paisley, United Kingdom) with capillary electrophoresis performed by Micromon, Monash University. The 66 HEVNet sequences (phyloanalysis population) were also analyzed phylogenetically.

### Genotype and Phylogenetic Analysis

Using the sequence analysis program SeqScape version 2.1.1 (Applied Biosystems, Life Technologies, Ltd, Paisley, United Kingdom), a consensus sequence was generated for all isolates by aligning the forward and reverse sequence strands of the amplicon of sample to the stored reference HEV genome sequences NC_001434 and AB248521 from Genbank. For phylogenetic analyses, HEVnet sequences obtained from the 66 patient samples (phyloanalysis group) were multi-sequence aligned with full-length HEV sequences downloaded from GenBank (Benson et al., 2014) using the MAFFT program (Katoh et al., 2002) implemented in the bioinformatics software platform Geneious v7.1.9^1^. The alignments were then manually trimmed to the same length of the amplicon sequences. Subtype confirmation and assessment of phylogenetic relationships between the HEV sequences were performed using Maximum Likelihood (ML) phylogenetic trees constructed in IQtree (Nguyen et al., 2015). The best-fit substitution model used for each sequence data set was determined using ModelFinder (Kalyaanamoorthy et al., 2017) based on Bayesian Information Criterion. Clade support was assessed using 1,000 pseudo-replicates generated with the UFBoot non-parametric bootstrap procedure (Hoang et al., 2018) implemented in IQ-TREE. The ML tree used to confirm HEV genotype of patient isolates determined by screening primers, was constructed using the GTR + F + R10 model by assessing their phylogenetic relationships with 594 reference HEV genome sequences from GenBank, including the proposed set of reference HEV sequences for each subtype (Smith et al., 2020).

Once the genotype was confirmed, a ML phylogenetic tree was generated separately for HEV genotypes 1, 3, and 4 with the sample isolates and four to five representative sequences for each subtype, using the TNe + I + G4 model for HEV-1, the TIM3e + I + G4 model for HEV-3 and the TIM2e + G4 model for HEV-4 as recommended by the ModelFinder (Kalyaanamoorthy et al., 2017) option on IQtree, with bootstrap replicates of 1000 using UFBoot (Hoang et al., 2018).

Sample isolates that were unable to be subtyped were uploaded to the HEVnet typing tool^2^ for further analysis.

The nucleotide sequences of this study were deposited in GenBank (Accession number MW355681 – MW355746).

## Results

### Laboratory Data

Between 2015 and 2020, a total of 909 serum or plasma samples were tested at VIDRL for HEV RNA with samples sent from laboratories across all states and territories except for QLD. Of these, 91 samples had detectable HEV RNA with 55 isolates identified as HEV-1 (60.4%); 34 (37.4%) as HEV-3, and 2 (2.2%) as HEV-4 based on the 191nt in-house PCR amplicon of ORF2.

### Serology Data

Anti-HEV IgG serological data was also available for 34/91 HEV RNA positive samples (data not shown), with 24/34 (71%) having a s/co value greater than 3. Eight samples had a s/co value less than 3 including four with a s/co value below 2. Two additional samples did not have detectable anti-HEV IgG at the time that HEV RNA was detected including one patient who was immunosuppressed.

### Genotyping and Phylogenetic Analyses

Sixty-six of the 91 HEV RNA positive samples were available for testing using the HEVnet protocol while 25 samples had insufficient serum for testing. Table 1 shows the demographic and clinical characteristics of these 66 cases (phyloanalysis population) including average patient age, travel history and genotype distribution with 42 (63.6%) isolates identified as HEV-1, 22 (33.3%) as HEV-3 and 2 as HEV-4 (3.0%). This is consistent with the genotype distribution of the 91 HEV RNA positive samples tested with the in-house protocol at VIDRL.

**TABLE 1:** Demographic and clinical history of HEV RNA positive cases, divided by genotype.

	**HEV-1**	**HEV-3**	**HEV-4**	**Total**
Number of isolates (% of total number)	42 (64%)	22 (33%)	2 (3%)	66
Median Age (range), years	30.6 (17-66.9)	61.7 (35-82.1)	63.5 (63.5)	
**Gender**				
Male	22 (52.4%)	11 (50.0%)	1 (50%)	34
Female	14 (33.3%)	8 (36.4%)	1 (50%)	23
Unknown	6 (14.3%)	3 (13.6%)	0	9
**State/Territory of Origin**				
Victoria	12 (28.6%)	3 (13.6%)	1 (50%)	16
NSW	15 (35.7%)	14 (63.6%)	1 (50%)	30
Western Australia	9 (21.4%)	4 (18.2%)	0	13
South Australia	4 (9.5%)	0	0	4
Tasmania	1 (2.4%)	0	0	1
ACT	0	1 (4.5%)	0	1
Northern Territory	1 (2.4%)	0	0	1
**Recent travel**				
India	20 (47.6%)	0	0	20
Pakistan	3 (7.1%)	0	0	3
China	0	0	1 (50%)	1
Japan	1 (2.4%)	0	0	1
Portugal	0	1 (4.5%)	0	1
England	0	1 (4.5%)		1
Singapore	0	1 (4.5%)		1
Undisclosed travel	2 (4.8%)	0	0	2
Unknown	16 (38.1%)	13 (59.1%)	1 (50%)	29
Locally acquired	0	6 (27.3%)	0	6

### HEV Genotype 1

**FIGURE 1:**
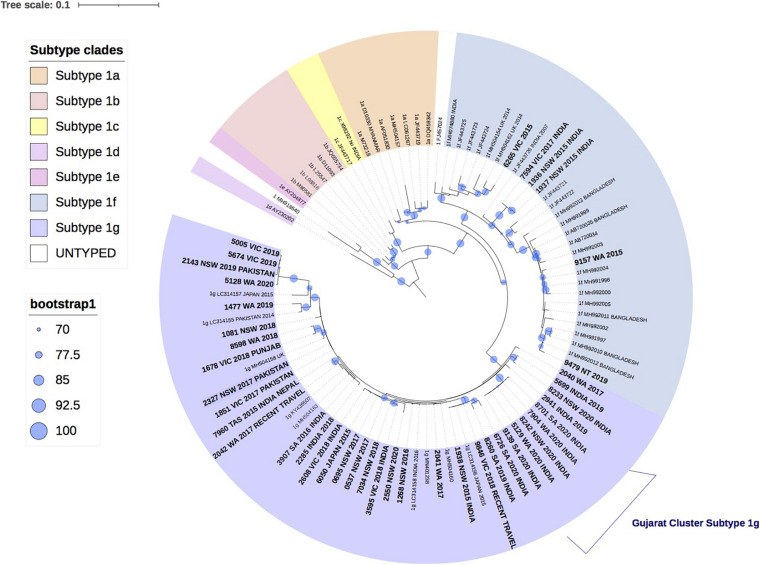
A maximum likelihood phylogenetic tree of the hepatitis E virus (HEV) ORF2 region (493nt) showing the inferred relationship between the HEV-1 sequences isolated from this study, and HEV sequences of known genotype 1 retrieved from Genbank. The tree was constructed using the model TNe + I + G4, and branches are annotated with UF bootstrap support (1,000 replicates). Significant bootstrap values (>70) are reported. Accession numbers and country of origin of the sequences downloaded from Genbank are indicated on the tree. Subtypes are indicated by color. The HEV sequences from this study are depicted in bold, with known country of origin or recent travel indicated.

Figure 1 shows the result of the phylogenetic analysis of 42 HEV-1 VIDRL isolates and the HEV-1 subtype references. This tree shows that the reference sequences are consistently grouped into the correct subtypes despite the short amplicon length. Phylogenetic analysis showed that six isolates clustered within the 1f subtype with bootstrap support of 99% but the majority of VIDRL isolates (*n* = 36/42, 86%) cluster with the subtype 1g with 97% bootstrap support. This includes a cluster of five sequences (100% bootstrap value) from unrelated individuals who had all traveled to Gujarat, India within a similar time period. However, most cases that are genetically related were identified from different years. More than 60% (*n* = 26/42) of patients diagnosed with HEV-1 reported a history of travel including 23/42 (54%) patients who had traveled to India or Pakistan.

### HEV Genotype 3

**FIGURE 2:**
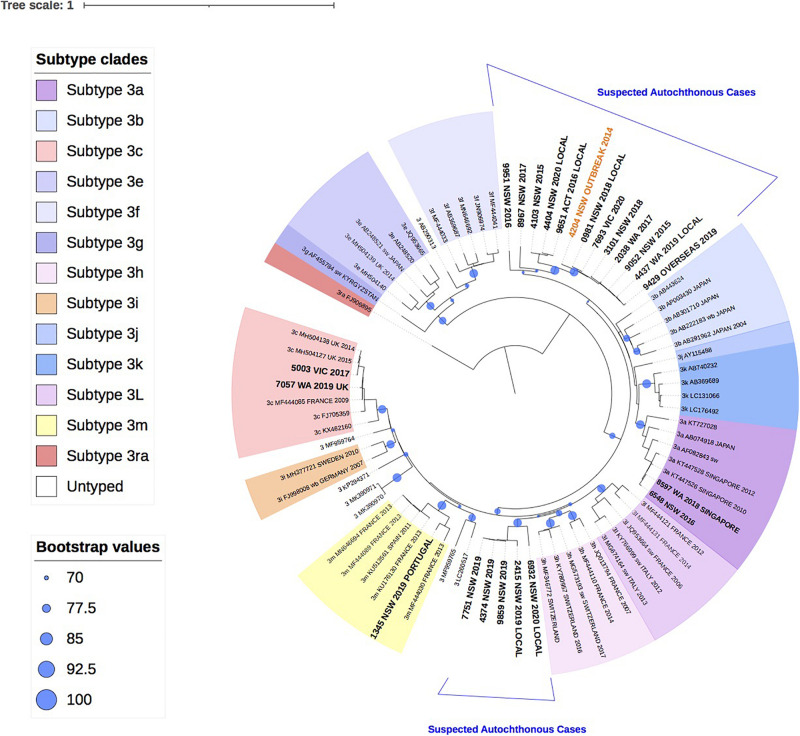
A maximum likelihood phylogenetic tree of the hepatitis E virus (HEV) ORF2 region (493nt) showing the inferred relationship between the HEV-3 sequences isolated from this study, and HEV sequences of known genotype 3 retrieved from Genbank. The tree was constructed using the model TIM3e + I + G4, and branches are annotated with UF bootstrap support (1,000 replicates). Significant bootstrap values (>70) are reported. Accession numbers and country of origin of the sequences downloaded from Genbank are indicated on the tree. Subtypes are indicated by color. The HEV sequences from this study are depicted in bold, with known country of origin indicated.

Figure 2 shows the phylogenetic relationship of 22 HEV-3 isolates with the reference HEV-3 subtypes. Despite the short amplicon length, the reference sequences clustered appropriately into subtype groups. All 22 study isolates clustered within HEV-3 group 2 (HEV-3 abchij) but only five isolates could be subtyped. Three of the five isolates clustered to subtypes that have been reported in European countries. Of these, two isolates were identified as subtype 3c, one of whom had a history of travel to the United Kingdom during the incubation period. The third isolate was identified as subtype 3m and the patient had reported recent travel to Portugal. The two remaining isolates that could be subtyped had clustered with 3a sequences previously reported in Singapore. One patient was confirmed to have traveled to Singapore in the incubation period.

Twelve isolates collected between 2015 and 2020 from patients in Western Australia (WA), Victoria (VIC), NSW and Australian Capital Territory (ACT), formed a unique clade in the HEV-3 group 2 (HEV-3 abchij) monophyletic group and could not be subtyped. This clade included the index case from the 2013/4 NSW HEV-3 outbreak that was linked to consumption of undercooked pork (Yapa et al., 2016). Two distinct clusters were observed in this clade. The first cluster had seven isolates (>98% bootstrap support) that shared a common ancestor with the NSW outbreak index strain and had pairwise sequence homology that ranged between 97.2 and 99.2%. Two of these patients were suspected to have locally acquired infection. A related second cluster of 3 isolates from NSW and ACT (100 bootstrap support) shared a common ancestor with the 2013/4 NSW HEV-3 outbreak sequence with 93.8–94.6% sequence identity. Two of these patients were suspected to have locally acquired infection.

Additionally, five isolates collected from NSW patients in 2019 and 2020 formed a second unique clade with a bootstrap value of 99% within the HEV-3abchij group. These isolates formed a sister clade with the European subtype 3h sequences, which has been reported in humans, pigs and wild boar (Adlhoch et al., 2009; Smith et al., 2020). Three of these isolates were collected within a 6-week period in NSW and shared 100% sequence homology. Whilst no clinical information was available for these cases, a fourth isolate suspected of being locally acquired, shared 97.4% homology with these sequences. A fifth isolate, collected from an NSW patient in 2020 and believed to be locally acquired, also clustered with this group with 90% sequence identity.

### HEV Genotype 4

**FIGURE 3:**
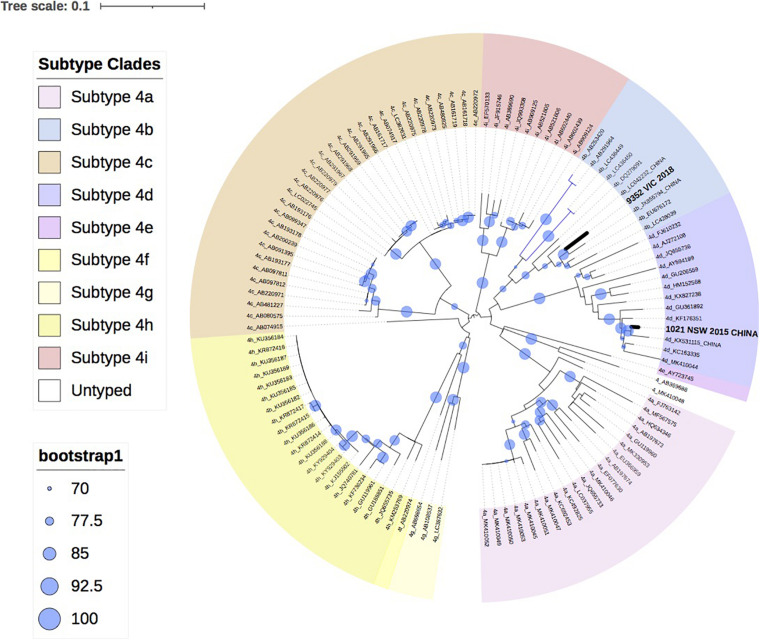
A maximum likelihood phylogenetic tree of the hepatitis E virus (HEV) ORF2 region (493nt) showing the inferred relationship between the HEV-4 sequences isolated from this study, and HEV sequences of known genotype 4 retrieved from Genbank. The tree was constructed using the TIM2e + G4 model and branches are annotated with UF bootstrap support (1,000 replicates). Significant bootstrap values (>70) are reported. Accession numbers and country of origin of the sequences downloaded from Genbank are indicated on the tree. Subtypes are indicated by color. The HEV sequences from this study are depicted in bold, with known country of origin indicated.

Figure 3 shows the two HEV-4 isolates cluster with subtypes 4b and 4d, respectively, with strong bootstrap support.

## Discussion

This is the first study to characterize the genotype distribution and perform phylogenetic analysis of HEV in Australia. The phyloanalysis demonstrates that the majority of HEV infections in Australia are associated with overseas travel (*n* = 48, 73%), however, autochthonous infections continue to occur annually. As expected, the majority of HEV infections tested at VIDRL were genotype 1 (*n* = 55, 60.4% of all isolates, 64% of the phyloanalysis group), which is prevalent in South Asian countries including India and Pakistan, where more than 54% (*n* = 23/42) of the phyloanalysis population with HEV-1 had reported recent travel. Whilst travel history was not provided for 16/42 (38%) of this group with HEV-1, the phylogenetic analysis indicates that these isolates are related to 1g or 1f sequences previously reported in India, Pakistan, and Japan (Nishizawa et al., 2017; Pathak and Barde, 2017). This is consistent with previous publications in which 88% of people diagnosed with hepatitis E in NSW had reportedly traveled to South Asia (Gunaratnam et al., 2014).

Phylogenetic analysis further revealed a cluster of five HEV-1 sequences, classified as subtype 1g, which shared 99.9% sequence identity and were collected from unrelated patients in South Australia (SA), WA and NSW, who had all traveled to Gujarat, India in 2019 and 2020. Outbreaks of hepatitis E infection are common in India and have been linked to faecal contaminated water, with subtypes 1a, 1c, and 1f most commonly identified (Gurley et al., 2014; Tripathy et al., 2019; Malhotra et al., 2020). HEV subtype 1g was first proposed as a new subtype in 2017 and has retrospectively been identified as the cause of a hepatitis E outbreak in Nepal and Mongolia in 2014 and 2015, respectively (Nishizawa et al., 2017).

Patients with HEV-1 were more likely to be younger with an average age of 30.6 years compared to 61.7 years for patients with HEV-3 as has previously been described (Kamar et al., 2017). Where gender was known, males were more likely to be infected, representing 61.1% of HEV-1 cases and 57.9% of HEV-3 cases. This is consistent with previous studies where symptomatic HEV-3 infections affect males in 75% of reported cases (Dalton et al., 2008). It is important to note that two of the female patients were receiving immunosuppressive therapy and another two women were >70 years of age. In our analysis, ten patients had only weakly reactive anti-HEV IgG, including two patients who were immunocompromised, so if HEV is clinically suspected, it is important to consider molecular testing even if the serological results are not indicative of current infection. IgG is used at VIDRL in favor of IgM, which is not recommended in settings where HEV infection is uncommon (Lin et al., 2000). However, it may have been useful in this case and its implementation may be considered. Nonetheless follow-up samples are routinely requested when the s/co is <3 to detect potential sero-converters.

In this report 34 (37.4%) (or 22, 33% phyloanalysis group) strains over the 6-year study period in Australia were classified as genotype 3. This is lower than that reported in other industrialized nations where HEV-3 is the dominant cause of hepatitis E infections including Belgium (92%), England (85%) and Bulgaria (98%) (Bruni et al., 2018; Oeser et al., 2019; Suin et al., 2019). Indeed, a 2016 investigation of HEV RNA prevalence in Australian blood donors was estimated to be 1 in 74,131, which was lower than the HEV RNA prevalence estimates reported by other developed countries (Hoad et al., 2017). Prior to 2013, there had only been one published case of locally acquired HEV-3 in Australia in 1995 (Heath et al., 1995), with a further two unpublished reports in 2005. In this analysis, there were potentially 17 cases of HEV-3 that were locally acquired, Figure 2, with at least six of these having reported a clinical suspicion of local acquisition. The phylogenetic analysis shows that these isolates formed two distinct clusters in the GT3 group 2 clade. These isolates could not be subtyped based on the reference sequences or by the HEVnet typing tool and may actually represent new “Australian” subtype/s. This certainly warrants further investigation.

As HEV-3 is considerably diverse the subtype classification continues to be updated and recent revisions have proposed 13 HEV-3 subtypes and six unclassified isolates (Smith et al., 2020). For a new subtype to be designated, at least three full-length HEV sequences are required from sources that are not epidemiologically linked. Whilst 17/22 (77%) of HEV-3 isolates in this study could not be subtyped, full genome analysis will be attempted in future analyses to ascertain if these isolates constitute new Australian subtype/s. Nonetheless, results here suggest that there are at least two genetically distinct HEV strains circulating in Australia. The first group of cases consisted of five HEV-3 isolates that were all collected in NSW within a 12-month period. Whilst three of these cases did not have travel history provided, they shared a common ancestor with two cases with suspected local acquisition. For the second group, ten of the HEV-3 study isolates shared a common ancestor with the index case of the 2013/4 NSW HEV outbreak, sharing 93.8–99.2% sequence identity. This supports our findings that these cases are likely to be autochthonous infections. There were another two isolates that were genetically related to this group, sharing 89.6–90.1% sequence identity with the outbreak sequence inferring that these cases may also have been locally acquired.

The 2013/4 HEV outbreak in NSW was linked to consumption of undercooked pork liver sourced in Australia (Yapa et al., 2016). Indeed, all fresh pork consumed in Australia is locally sourced and imported pork products must be cooked to inactivate any pig diseases (Australian Pig Farmers, 2020). Previous studies, in China and the Nordic region of Europe, have shown high similarities between HEV strains isolated from patients and pigs in the same geographical region (Norder et al., 2009; Fu et al., 2010). In our study, 13/17 (76%) of suspected locally acquired HEV-3 infections occurred in patients from NSW and ACT, which was a surprising observation given that NSW produces only 16% of the total number of Australian pigs annually (Australian Pork Limited, 2019). It is not known if NSW is over-represented in autochthonous HEV-3 cases due to a higher incidence of HEV circulating in NSW pigs or perhaps due to enhanced surveillance for locally acquired hepatitis E following the 2013/4 outbreak. Interestingly, in a 1999 seroprevalence study of anti-HEV IgG in Australian pigs, four of six farms included were in NSW, including two where 90–95% of pigs were seropositive for HEV antibodies by 16 weeks of age (Chandler et al., 1999). A research pig farm from VIC was used as a HEV negative control in this study and wild pigs from the Northern Territory (NT) were also sampled but no pig farms from other states or territories were included. As such, further investigation is needed to ascertain the current prevalence of HEV in pigs across Australia as well as characterizing the circulating HEV strains.

In this analysis, only 5/22 (23% phyloanalysis group) HEV-3 cases were believed to have been acquired overseas based on travel history and phylogenetic analysis. Two isolates, that were collected 2 years apart and from different states, were subtyped as 3a based on phylogenetic analysis and clustered with sequences reported in Singapore. This concurs with the travel history of one of these patients who had traveled to Singapore within the incubation period. HEV genotype 3a strains have been isolated from patients and pigs in Singapore, where HEV-3 is the dominant genotype (Wong et al., 2019). Similarly, two isolates were identified as subtype 3c, which has been reported in European countries including France and England and was consistent with the travel history reported for one of the patients. Interestingly, the second patient was chronically infected with HEV and had previously received a liver transplant. No travel history was available for this patient and we cannot rule out that the infection was acquired through transplantation or transfusion, which has previously been described (Speers et al., 2015). Alternatively, HEV infection may have contributed to the patient’s liver failure prior to transplantation.

Finally, this analysis showed that infection with HEV-4 is rare in Australia, with two cases in the last 5 years. This is consistent with industrialized countries in Europe, where HEV-4 has been infrequently reported, such as Belgium (<2%), England (<1%) and Switzerland (3.6%) (Fraga et al., 2018; Oeser et al., 2019; Suin et al., 2019). In this study, phylogenetic analysis indicates that both isolates clustered with sequences identified in China, which is consistent with the travel history of one of the patients. HEV-4 is largely confined to Asia and a study in Eastern China found that 93.5% of hepatitis E infections were caused by the zoonotic HEV-4 (Lu et al., 2006; Zhu et al., 2014).

A limitation of this study is that it only represents HEV positive samples that were tested at VIDRL. No samples from QLD were analyzed and NSW laboratories were most likely to send samples that were suspected of being locally acquired as recommended by the Department of Health. QLD only reported the first published case of locally acquired HEV-3 in 2019 (Taylor et al., 2020). As such, the incidence of HEV-3 may be over-represented in this analysis. Nonetheless, this study has shown that small numbers of locally acquired infections continue to occur annually and it is important to screen for HEV infections in patients presenting with unexplained hepatitis with and without travel history.

## Conclusion

This study characterizes the HEV isolates detected in Australia thereby contributing to the global understanding of autochthonous HEV-3 and confirming the acquisition of HEV-1 during travel to endemic areas. Results also suggest the presence of a new “Australian” subtype/s of HEV-3 which requires further investigation. This provides valuable information to clinicians and scientists with respect to diagnosis of HEV infection and may prepare us to implement public health measures should an outbreak occur. The morbidity and mortality associated with HEV infection, particularly in immunosuppressed individuals and pregnant women, reaffirms the need to screen for HEV both locally and in travelers.

## Data Availability Statement

The nucleotide sequences of this study were deposited in GenBank (Accession numbers MW355681–MW355746).

## Author Contributions

JO’K: data curation, formal analysis, investigation, methodology, and roles/writing – original draft. LT: methodology. LY: formal analysis and writing – review and editing. SB and XL: methodology. BC: investigation and writing – review and editing. SN: methodology and writing – review and editing. KJ: conceptualization, project administration, supervision, and writing – review and editing. All authors contributed to the article and approved the submitted version.

## Conflict of Interest

The authors declare that the research was conducted in the absence of any commercial or financial relationships that could be construed as a potential conflict of interest.

## Footnotes

1 https://www.geneious.com

2 https://www.rivm.nl/mpf/typingtool/hev/
